# Is Tadalafil an Effective Treatment Option for Interstitial Cystitis/Painful Bladder Syndrome? A Report of a Challenging Case

**DOI:** 10.7759/cureus.16717

**Published:** 2021-07-29

**Authors:** Abdullah Demirtaş, Gökhan Sönmez, Şevket T Tombul, Türev Demirtaş

**Affiliations:** 1 Urology, Erciyes University, Kayseri, TUR; 2 History of Medicine and Ethics, Erciyes University, Kayseri, TUR

**Keywords:** tadalafil, bladder pain syndrome, interstitial cystitis, chronic pelvic pain, pde 5 inhibitor

## Abstract

Interstitial cystitis/painful bladder syndrome (IC/PBS) is a chronic pelvic pain (CPP) syndrome that is frequently seen in female patients. Since its molecular mechanism and etiopathogenesis are not clearly elucidated, its treatment options are limited. Phosphodiesterase-5 (PDE-5) inhibitors act on nitric oxide (NO) and cyclic guanosine monophosphate (cGMP) and are an effective treatment option in some CPP syndromes. We discuss the case of a 44-year-old female patient who presented to our clinic with a two-year history of frequent urination and pain in the pelvic area. The cystoscopy of the patient, who did not benefit from first- and second-line treatments, was normal. With the diagnosis of IC/PBS, she was started on tadalafil (oral) 5 mg/day. At the end of a total of 12 months of follow-up, it was observed that the patient's symptoms significantly regressed.

Based on our findings, the relaxing effect of PDE-5 inhibitors on the bladder neck/detrusor muscle and the vasodilator effect on the blood supply to the pelvic organs may have improved the patient's symptoms. In this case report, for the first time in the literature, we present the clinical outcomes of treatment with tadalafil (5 mg/day), which is a PDE-5 inhibitor, in a female patient with IC/PBS who did not respond to first-and second-line treatments. The results indicated that tadalafil, which shows activity through the NO-cGMP and prostaglandin pathway, is a potential alternative in IC/PBS patients resistant to conventional first- and second-line treatments.

## Introduction

Chronic pelvic pain (CPP) is defined as persistent noncyclic pain that is perceived to be in the pelvic area. Irritable bowel syndrome (IBS), endometriosis, and interstitial cystitis/painful bladder syndrome (IC/PBS) are the most common subtypes of CPP [1].

IC/PBS is a chronic painful bladder disease characterized by pelvic pain and urinary symptoms in the absence of any other identifiable pathology. Although it may occur due to pathologies such as glomeration and Hunner’s ulcers, the etiology is not clearly known in most cases. Although its prevalence among women is remarkably high, it is reported to be between 2.7-6.5% in the USA. Due to the lack of consensus on its definition and etiology, the proposed treatment methods have wide variations [2]. Professional guidelines recommend analgesics, lifestyle changes, and patient education as first-line treatment and the use of intravesical treatments (lidocaine, heparin) and oral therapies such as amitriptyline, cimetidine, and hydroxyzine as second-line treatment. In patients who do not benefit from these treatments, much more invasive methods such as cystoscopy-fulguration, cystoscopy-hydrodistention, botulinum toxin A, neuromodulation, or even cystectomy with diversion are preferred [1].

Phosphodiesterase-5 (PDE-5) inhibitors lead to the increase of nitric oxide (NO) and cyclic guanosine monophosphate (cGMP) levels, resulting in the relaxation of the bladder neck, inhibition of neurogenic contractions in the bladder neck, and inhibition of vasoconstriction caused by prostaglandins and ultimately result in successful pain relief. Moreover, by means of a similar mechanism, the tissue remains relaxed and the blood circulation in the tissue is enhanced [3]. Based on this mechanism of action, many studies have suggested that PDE-5 inhibitors are effective in treating CPP. Tadalafil and sildenafil are among the most commonly used PDE-5 inhibitors [4,5].

In this case report, for the first time in the literature, we present the clinical outcomes of treatment with tadalafil, a PDE-5 inhibitor, in a female patient who had IC/PBS and did not respond to first- and second-line treatments.

## Case presentation

A 44-year-old otherwise healthy female patient, with a body mass index of 27.2 kg/m^2^, presented to our clinic with a two-year history of frequent urination and pain in the pelvic area. The patient reported that the pain in the suprapubic region increased while urinating and also at regular intervals during the day. It was also revealed that the patient did not benefit from lifestyle changes, various analgesics, oral hydroxyzine treatment, and anticholinergic (oxybutynin and trospium) treatment. A cystoscopy performed at another healthcare center had detected no pathology in the bladder and the random biopsy results were benign. Additionally, the patient also did not benefit from the hydrodistention performed in the same session with cystoscopy. On physical examination, urogenital findings were normal. No cystocele, rectocele, or descensus was detected and the stress test was negative. There was no pathology in the gynecological evaluation of the premenopausal patient. Urinalysis was normal and showed no growth in the urine culture. In uroflowmetry, the maximum flow rate was 32 ml/sec, the voided urine volume was 120 ml, and no post-voiding residual urine was detected. Data from the three-day voiding diary showed that the patient urinated every 30 min. The mean daily fluid intake during the three days was 2600, 3100, and 2900 ml, respectively. Bladder functions were normal in the flow-pressure test performed with the suspicion of an overactive bladder.

**Figure 1:**
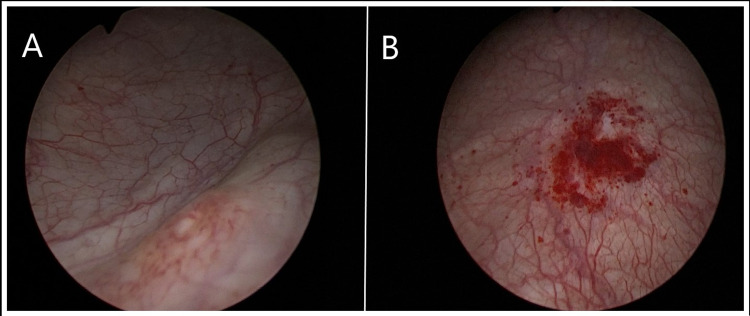
Cystoscopy images A. Normal cystoscopic findings and left orifice. B. Random punch biopsy area

**Figure 2:**
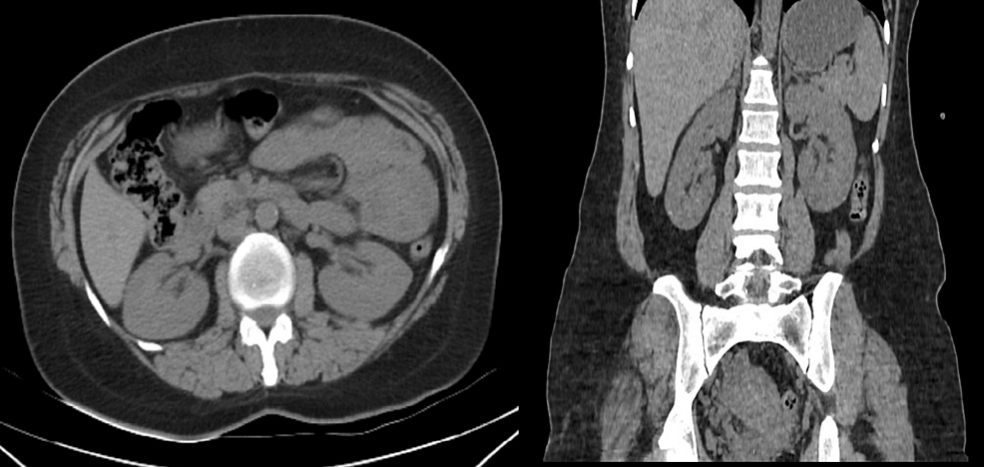
CT abdomen revealed no abnormal findings CT: computed tomography

The patient completed The Impact of Female Chronic Pelvic Pain Questionnaire (IF-CPPQ) and had a score of 76. Bladder walls and orifices were normal on repeat cystoscopy and there was no pathology in the bladder (Figure 1A). The histopathology of random punch biopsy taken from the bladder was reported as "chronic inflammatory changes" (Figure 1B). Abdominal CT showed no urological or pelvic pathology that could cause pelvic pain (Figure 2). The patient was prediagnosed as having IC/PBS and was initiated on oral tadalafil 5 mg once daily (two hours after breakfast). At each four-week follow-up visit, the symptoms decreased significantly. There were no drug-related side effects. At the 12-week follow-up, the voiding intervals were prolonged and the number of daily micturition decreased to 10 although the volume of daily fluid intake was similar to that of the pre-treatment period. Additionally, the pain during urination disappeared significantly and the post-treatment IF-CPPQ score was 40. Tadalafil treatment was discontinued at the end of six months. No side effects of tadalafil were observed and no relapse occurred during the 12-month follow-up.

## Discussion

To our knowledge, this is the first case report to evaluate the clinical outcomes of daily tadalafil treatment in a female patient with IC/PBS based on the relaxation effect of PDE-5 inhibitors on the bladder neck/detrusor muscle and its vasodilator effect on the blood supply to the pelvic organs. Although the patient was resistant to the step therapies recommended by the guidelines, the tadalafil therapy led to a significant reduction in the symptoms. Based on our results, we suggest that the PDE-5 inhibitors acting through the prostaglandin and NO-cGMP pathway may have a common mechanism with IC/PBS, whose exact etiopathogenesis remains unknown.

Previous studies have indicated that the PDE-5 inhibitors might be useful in the treatment of CPP [3-5]. Benelli et al. administered oral tadalafil 5 mg once daily in 20 male patients with chronic prostatitis/chronic pelvic pain syndrome (CP/CPPS) and reported that the symptoms improved in approximately 70% of the patients [4]. In contrast, Russell et al. reported that tadalafil led to a significant reduction in bladder symptoms and pain in a female patient with loin pain-hematuria syndrome (LPHS) [5]. In a randomized prospective study, Dmitrovic et al. showed that sildenafil, a PDE-5 inhibitor, led to a significant reduction in the symptoms of CPP associated with dysmenorrhea [3]. Our patient, unlike those reported in other studies, underwent long-term daily tadalafil treatment due to IC/PBS and the symptoms resolved significantly. Based on our findings, we consider that PDE-5 inhibitors may be effective in IC/PBS, which is a common subtype of CPP.

## Conclusions

The results indicated that tadalafil, which shows activity through the NO-cGMP and prostaglandin pathway, is a potential alternative in resistant IC/PBS patients. Further randomized controlled and prospective studies are needed to elucidate the etiopathogenesis and molecular mechanisms of IC/PBS.
